# Submicroscopic copy-number variations associated with 46,XY disorders of sex development

**DOI:** 10.1186/s40348-015-0018-2

**Published:** 2015-04-30

**Authors:** Masafumi Kon, Maki Fukami

**Affiliations:** Department of Molecular Endocrinology, National Research Institute for Child Health and Development, 2–10–1 Okura, Setagaya Tokyo, 157-8535 Japan; Department of Urology, Hokkaido University Graduate School of Medicine, North-15, West-7, Kita-ku Sapporo, 060-8638 Japan

**Keywords:** Disorders of sex development, Copy-number variations, Multiplex ligation-dependent probe amplification, Comparative genomic hybridization

## Abstract

**Background:**

Mutations in known causative genes and cytogenetically detectable chromosomal rearrangements account for a fraction of cases with 46,XY disorders of sex development (DSD). Recent advances in molecular cytogenetic technologies, including array-based comparative genomic hybridization (aCGH) and multiplex ligation-dependent probe amplification (MLPA), have enabled the identification of copy-number variations (CNVs) in individuals with apparently normal karyotypes.

**Findings:**

This review paper summarizes the results of 15 recent studies, in which aCGH or MLPA were used to identify CNVs. Several submicroscopic CNVs have been detected in patients with 46,XY DSD. These CNVs included deletions involving known causative genes such as *DMRT1* or *NR5A1*, duplications involving *NR0B1*, deletions involving putative cis-regulatory elements of *SOX9*, and various deletions and duplications of unknown pathogenicity.

**Conclusions:**

The results of recent studies highlight the significance of submicroscopic CNVs as the genetic basis of 46,XY DSD. Molecular cytogenetic analyses should be included in the diagnostic workup of patients with 46,XY DSD of unknown origin. Further studies using aCGH will serve to clarify novel causes of this condition.

## Introduction

46,XY disorders of sex development (46,XY DSD) are clinically and genetically heterogeneous conditions that lead to genital abnormalities at birth, defective sexual development during puberty, and infertility in adulthood [1,2]. To date, mutations in several genes have been identified in patients with 46,XY DSD. Known causative genes for 46,XY DSD include *ATRX*, *CBX2*, *DHH*, *DMRT1*, *GATA4*, *MAP3K1*, *NR0B1* (alias *DAX1*), *NR5A1* (alias *SF1*), *RSPO1*, *SOX9*, *SRY*, *WNT4*, and *WT1* involved in testicular development; *AKR1C2/4*, *AR*, *CYP11A1*, *CYP17A1*, *LHCGR*, *HSD3B2*, *HSD17B3*, *POR*, *SRD5A2*, and *STAR* involved in androgen production or function; and *AMH*, *AMHR2*, *INSL3*, *RXFP2*, and the *HOXD* cluster involved in genital organ formation [1,3,4]. In addition, various chromosomal rearrangements have also been associated with 46,XY DSD [1,3]. However, mutations in known causative genes and cytogenetically detectable chromosomal rearrangements have been identified in only 20% to 30% of cases [1], indicating that other genetic or environmental factors play an important role in the development of 46,XY DSD.

Recent advances in molecular technology, including array-based comparative genomic hybridization (aCGH), multiplex ligation-dependent probe amplification (MLPA), and next-generation sequencing (NGS), have enabled high-throughput analysis of clinical samples. Of these, aCGH is highly useful to detect copy-number variations (CNVs) in the genome of individuals with apparently normal karyotypes [5,6], and MLPA can identify various copy-number alterations in specific disease-associated loci [7]. NGS primarily focuses on identification of nucleotide substitutions. Molecular cytogenetic analyses using aCGH or MLPA revealed the importance of CNVs as the cause of several genetic disorders, although accumulating evidence shows that submicroscopic CNVs can also occur as functionally neutral polymorphisms [6]. Here, we review recent reports on molecular cytogenetic analyses of patients with 46,XY DSD.

## Findings

### 46,XY DSD-associated CNVs identified by molecular cytogenetic analyses

In this review, we summarize the results of 15 recent studies [8-22]. We found these papers through a PubMed search using the key words ‘disorders of sex development’, together with ‘comparative genomic hybridization’, ‘multiplex ligation-dependent probe amplification’, or ‘copy-number variations’. We focused on original articles, in which aCGH or MLPA was used to identify submicroscopic CNVs in patients with 46,XY DSD. The 15 studies showed 28 deletions and 4 duplications as genetic causes of 46,XY DSD, as well as several other CNVs whose association with the disease phenotype remains uncertain (Table 1, parts a and b, and Table 2) [8-22]. Notably, White et al. identified pathogenic CNVs in 3 of 23 patients with 46,XY gonadal dysgenesis [18], and Igarashi et al. detected CNVs in 3 of 24 patients with various types of 46,XY DSD [9]. These data suggest the significance of submicroscopic CNVs in the etiology of 46,XY DSD. All CNVs, except for those on the sex chromosomes, were detected in the heterozygous state. Parental samples of the CNV-positive patients were analyzed in some cases, confirming the *de novo* occurrence or maternal inheritance of all CNVs examined (Table 1, parts a and b).

**Table 1:** **46,XY DSD-associated deletions identified by molecular cytogenetic analyses**

	**Case/family**	**Patient**	**Size of deletion**	**Locus**	**Affected DSD-causative genes**	**Gonadal phenotype**	**External genitalia**	**Additional clinical features**	**Method**	**Inheritance**	**Reference**
a) Deletions encompassing known 46,XY DSD-causative genes											
	Case 1		74 kb	2p16.3	*LHCGR*	Testes with rete testis, epididymic structures, residual adrenal structures	Ambiguous genitalia	N.D.	aCGH	*De novo*	Richard et al. [8]
	Case 2		8.5 Mb	9p24.1-p24.3	*DMRT1*	Streak gonads with ovarian ducts	Female	MR, schizophrenia	aCGH	N.D.	Igarashi et al. [9]
	Case 3		10.6 Mb	9p23-p24.3	*DMRT1*	PGD with dysgerminoma	Clitoromegary	Mild MR	aCGH	N.E.	Ledig et al. [10]
	Case 4		9.7 Mb	9p23-p24.3	*DMRT1*	GD	N.D.	Hydrops, facial dysmorphism, limb and kidney abnormalities	aCGH	*De novo*	Ledig et al. [10]
	Case 5		821.6 kb	9p24.3	*DMRT1*	CGD	Female	None	aCGH	N.E.	Ledig et al. [10]
	Case 6		103.2 kb	9p24.3	*DMRT1*	CGD	Female	None	aCGH	N.E.	Ledig et al. [10]
	Case 7		9.7 Mb/duplication (26 Mb)	9p23-p24.3/9p13.1-p23	*DMRT1*	GD	N.D.	None	Targeted aCGH	*De novo*	Tannour-Louet et al. [11]
	Case 8		6.7 Mb	9p24.1-pter	*DMRT1*	GD	N.D.	None	Targeted aCGH	*De novo*	Tannour-Louet et al. [11]
	Case 9		0.26 Mb	9p24.3	*DMRT1*	CGD	N.D.	None	Targeted aCGH	N.D.	Tannour-Louet et al. [11]
	Case 10		0.20 Mb	9p24.3	*DMRT1*	GD	N.D.	None	Targeted aCGH	N.E.	Tannour-Louet et al. [11]
	Case 11		0.24 Mb	9q33.3	*NR5A1* (*SF1*)	Abnormal germ cells, no Leydig cells	Ambiguous genitalia	N.D.	aCGH	Maternal	Harrison et al. [12]
	Case 12		3.1 to 4.8 kb	9q33.3	*NR5A1* (*SF1*)	Leydig cell hyperplasia, scarce germ cells, carcinoma *in situ*	Female without uterus, clitoromegaly	N.D.	Custom MLPA	N.D.	Barbaro et al. [13]
	Case 13		0.96 Mb	9q33.3	*NR5A1* (*SF1*)	N.D.	Clitoromegaly, shallow vaginal entrance	Ptosis	aCGH	*De novo*	van Silfhout et al. 2009 [14]
	Case 14		1.54 Mb	9q33.3	*NR5A1* (*SF1*)	N.E.	Female	Mild MR, minor dysmorphisms	aCGH	*De novo*	Brandt et al. [15]
	Case 15		3.07 Mb	9q33.3-q34.11	*NR5A1* (*SF1*)	Ovotestis	Clitoromegaly	Genitopatellar syndrome	aCGH	N.D.	Schlaubitz et al. [16]
	Case 16		10 Mb	11p12-p14.1	*WT1*	N.E.	Female	WAGR syndrome^a^	aCGH	*De novo*	Le Caignec et al. [17]
	Case 17		18.0 Mb	2q31.1-q32.1	*HOXD* cluster	N.D.	Severe micropenis, hypospadias	Short stature, MR, multiple anomalies	aCGH	N.D.	Igarashi et al. [9]
	Case 18		503.2 kb	Yq11.223	AZFb-c region	CGD	Female	None	aCGH	N.D.	Ledig et al. [10]
b) Deletions in the upstream region of known 46,XY DSD-causative genes											
	Case 19		1.193 Mb	17q24.2-q24.3	Upstream of SOX9	CGD	Female	Cleft palate, short stature	SNP array	N.D.	White et al. [18]
	Case 20		3.3 Mb	17q24.2-q24.3	Upstream of SOX9	CGD	Female	Acampomelic campomelic dysplasia, kyphoscoliosis	aCGH	N.D.	Ledig et al. [10]
	Family 1	Proband	240 kb	17q24.3	Upstream of SOX9	Small testis (right), streak gonad (left)	Asymmetric external genitalia, urogenital sinus with a phallus	None	MLPA/aCGH	Maternal	Benko et al. [19]
	Family 1	Cousin	240 kb	17q24.3	Upstream of SOX9	Streak gonad with gonadoblastoma (right), ovary (left)	Female	None	MLPA/aCGH	Maternal	Benko et al. [19]
	Case 21		236.3 kb	17q24.3	Upstream of SOX9	Streak gonad (right), gonadal tumor (left)	Female	None	MLPA/aCGH	Maternal	Kim et al. [20]
	Family 2	Proband	65.4 kb	17q24.3	Upstream of SOX9	Dysgenic testis^b^	Ambiguous genitalia	None	MLPA/aCGH	Maternal	Kim et al. [20]
	Family 2	Sibling	65.4 kb	17q24.3	Upstream of SOX9	Dysgerminoma (right), streak gonad with a gonadoblastoma (left)	Female	None	MLPA/aCGH	Maternal	Kim et al. [20]
	Family 3	Proband	136 kb	17q24.3	Upstream of SOX9	Dysgenic gonad with a gonadoblastoma	Female	N.D.	MLPA/aCGH	Maternal	Kim et al. [20]
	Family 3	Maternal relative	136 kb	17q24.3	Upstream of SOX9	Ovarian dysgerminoma	Female	N.D.	MLPA/aCGH	Maternal	Kim et al. [20]
	Family 4	Proband	576.9 kb	17q24.3	Upstream of SOX9	CGD	Female	None	MLPA/aCGH	Maternal	Kim et al. [20]
	Family 4	Sibling	576.9 kb	17q24.3	Upstream of SOX9	CGD	Female	None	MLPA/aCGH	Maternal	Kim et al. [20]
	Case 22		35 kb	8p23.1	Upstream of GATA4	CGD	Female	Adrenal hypoplasia congenita	SNP array	N.D.	White et al. [18]
	Case 23		0.22 Mb	8p23.1	Upstream of GATA4	PGD	Ambiguous genitalia	N.D.	aCGH	Maternal	Harrison et al. [12]
	Case 24		257 kb	Xp21.2	Upstream of NR0B1 (DAX1)	Testis-like (right), streak gonad (left)	Female	None	aCGH	Maternal	Smyk et al. [21]

DSD, disorders of sex development; PGD, partial gonadal dysgenesis; CGD, complete gonadal dysgenesis; GD, gonadal dysgenesis of unknown severity; N.E., not examined; N.D., not described; MR, mental retardation; aCGH, array-based comparative genomic hybridization; MLPA, multiplex ligation-dependent probe amplification. ^a^Wilms tumor, aniridia, genitourinary anomalies, and mental retardation syndrome. ^b^Dysgenic testis with remnants of ducti deferentes and rete testis and with primitive seminiferous tubules.

**Table 2:** **46,XY DSD-associated duplications identified by molecular cytogenetic analyses**

**Case**	**Size of duplication**	**Locus**	**Affected DSD-causative gene**	**Gonadal phenotype**	**External genitalia**	**Other clinical features**	**Method**	**Reference**
Case 1	16.23 Mb	Xp21.1-p22.2	*NR0B1* (*DAX1*)	CGD	Female	IUGR, facial dysmorphism, disturbance of pulmonary adaption, muscular hypertonia, hearing defect, mental retardation, short stature, macrocephaly	aCGH	Ledig et al. [10]
Case 2	729 kb	Xp21.2	*NR0B1* (*DAX1*)	PGD with testicular residues	Clitoromegary	None	aCGH	Ledig et al. [10]
Case 3	771 kb	Xp21.2	*NR0B1* (*DAX1*)	CGD	Female	None	SNP array	White et al. [18]
Case 4	800 kb	Xp21.2	*NR0B1* (*DAX1*)	Streak gonad, testicular tissue with atrophic tubules (right)	Ambiguous genitalia	N.D.	Custom MLPA	Barbaro et al. [22]

DSD, disorders of sex development; PGD, partial gonadal dysgenesis; CGD, complete gonadal dysgenesis; N.D., not described; MLPA, multiplex ligation-dependent probe amplification; aCGH, array-based comparative genomic hybridization; IUGR, intrauterine growth restriction.

### Deletions encompassing known 46,XY DSD-causative genes

Submicroscopic deletions encompassing known causative genes were identified in 18 patients (Table 1, part a). These deletions ranged from 74 kb to 18.0 Mb and caused haploinsufficiency of *LHCGR*, *DMRT1*, *NR5A1*, *WT1*, or *HOXD* cluster [8-17]. Of these deletions, those encompassing *DMRT1* or *NR5A1* were relatively common. Notably, CNVs in cases 3, 16, and 17 were 10 to 18 Mb in length. These results imply that in some cases, deletions ≥10 Mb can be missed by standard karyotyping, although this method is expected to detect CNVs ≥5 to 10 Mb [11]. Actually, the results of karyotyping are affected by the quality of samples and the genomic position of CNVs [11]. Submicroscopic deletions in the 18 patients were associated with both isolated and syndromic 46,XY DSD. Syndromic DSD resulting from these deletions were primarily ascribed to contiguous gene deletions. For example, a deletion in case 16 encompassing *WT1* and *PAX6* caused 46,XY DSD, Wilms tumor, aniridia, and mental retardation, which is collectively referred to as WAGR syndrome. This WAGR syndrome has been described in several patients with cytogenetically detectable deletions at 11p. The size of submicroscopic deletions in 18 patients roughly corresponded to patients’ phenotypes (isolated or syndromic); 7 of the 9 patients with deletions ≥1.0 Mb manifested additional clinical features such as mental retardation, short stature, and facial dysmorphism, whereas such features were not reported in 9 patients with smaller deletions (Table 1, part a). On the other hand, syndromic 46,XY DSD was also caused by deletions of a single gene with complex functions. Indeed, finger anomalies observed in DSD patients with *HOXD* cluster-containing deletions are consistent with the fact that *HOXD* genes, particularly *HOXD13* [23], control the development of limb and external genitalia [4]. Although submicroscopic deletions involving known causative genes were frequently identified in patients with syndromic 46,XY DSD (Table 1, part a), previous molecular cytogenetic analyses may be biased toward patients with complex phenotypes. Thus, further studies are necessary to clarify the precise frequency of pathogenic CNVs in patients with isolated and syndromic 46,XY DSD.

### Deletions in the upstream regions of known 46,XY DSD-causative genes

Submicroscopic deletions that reside adjacent to known causative genes also underlie 46,XY DSD. To date, deletions involving the upstream regions of *SOX9*, *GATA4*, or *NR0B1* have been reported to cause 46,XY gonadal dysgenesis (Table 1, part b) [10,12,18-21]. These deletions are predicted to disrupt the cis-regulatory machinery of genes involved in testis formation. For example, SOX9 is regulated by SRY and plays a critical role in the development of testis and long bones [1]. *SOX9* abnormalities lead to 46,XY gonadal dysgenesis with or without campomelic dysplasia [1]. It is known that *SOX9* expression is tightly regulated by multiple cis-acting enhancers in the upstream and downstream regions and that elimination of the enhancer(s) leads to tissue-specific dysregulation of *SOX9* [10,18-20]. Previous studies have mapped *SOX9* enhancers for craniofacial tissues to a genomic interval >1.0 Mb apart from the coding region and a testis enhancer to a 32.5-kb region at a position 607 to 640 kb upstream from the start codon [10,18-20]. These enhancer regions seem to contain transcription factor binding sites which are essential to maintain *SOX9* expression during development. Since microdeletions in the *SOX9* upstream region can result in complete gonadal dysgenesis similar to that observed in patients with *SOX9* amorphic mutations, elimination of the distal enhancer seems to completely abolish *SOX9* expression on the affected allele [10,18-20]. Deletions in the upstream regions of *GATA4* and *NR0B1* are also likely to encompass cis-acting enhancers of these genes [12,18,21]. Identification of deletions in the upstream or downstream regions of genes provides clues regarding cis-regulatory mechanisms of each gene.

### Duplications encompassing known 46,XY DSD-causative genes

Submicroscopic duplications involving *NR0B1* were identified in several patients with 46,XY DSD (Table 2) [10,18,22]. *NR0B1* is a transcription factor gene isolated from the dosage-sensitive sex reversal adrenal hypoplasia congenita critical region on Xp21 [24]. Overdosage of *NR0B1* is a well-known cause of syndromic 46,XY DSD in individuals with large X chromosomal rearrangements [25]. Recently, molecular cytogenetic analysis by Smyk et al. has shown that submicroscopic duplications encompassing *NR0B1* can lead to 46,XY DSD as a sole clinical manifestation [21]. Copy-number gains of *NR0B1* were predicted to perturb testicular development by downregulating the protein expression of SF1, WT1, and SOX9 [18].

### CNVs of unknown pathogenicity

Genome-wide copy-number analyses identified several submicroscopic CNVs that could be associated with the development of 46,XY DSD. Ledig et al. performed aCGH analysis on 87 patients with 46,XY DSD and identified 31 CNVs which have not been described as polymorphisms [10]. While 7 of the 31 CNVs encompassed known DSD-causative genes, the other CNVs have not been implicated in DSD. Ledig et al. proposed that several genes, including *GKAP1*, *NCOA4*, and *CTNNA3*, are novel candidate genes for 46,XY DSD. Furthermore, Tannour-Louet et al. [11] analyzed 116 patients with 46,XY and 46,XX DSD and 8,951 control individuals, and identified 25 CNVs that may underlie DSD. Of these, 13 CNVs were detected in individuals with 46,XY karyotype. Further studies on large cohorts will clarify the pathogenicity of these CNVs.

### Clinical applications of molecular cytogenetic technologies

Molecular cytogenetic analyses using aCGH or MLPA would be beneficial for patients with 46,XY DSD, because identification of pathogenic CNVs could help to predict the disease outcome and possible complications of patients. For example, patients with deletions encompassing *WT1* have a high risk of Wilms tumor and renal failure. Furthermore, detection of disease-associated CNVs significantly improves the accuracy of genetic counseling for patients’ families. Molecular cytogenetic analyses, together with mutation screening using NGS, should be included in the diagnostic workup of patients with 46,XY DSD of unknown origin.

### Summary and conclusions

Recent studies revealed that submicroscopic CNVs constitute a fraction of the genetic causes of both isolated and syndromic 46,XY DSD. ACGH and MLPA appear to be useful for molecular diagnosis of patients with 46,XY DSD. Furthermore, genome-wide copy-number analyses using aCGH will serve to identify novel causes of 46,XY DSD.

## Abbreviations

aCGH: array-based comparative genomic hybridization
CNV: copy-number variation
DSD: disorders of sex development
MLPA: multiplex ligation-dependent probe amplification
